# Unusual Case of a Parasitic Extra-Uterine Leiomyoma Presenting With Lower Abdominal Pain

**DOI:** 10.7759/cureus.30141

**Published:** 2022-10-10

**Authors:** Georgia Micha, Dionysios G Galatis, Konstantina Kalopita, Antonios Strongylos, Christos Benekos, Konstantina Kalaitzi, Panagiotis-Konstantinos Karachalios, Foteini Anifantaki, Ioannis Dalivigkas, Ioannis Gripiotis, Nikolaos Kiriakopoulos, Argyrios Monastiriotis

**Affiliations:** 1 Department of Anesthesiology, Helena Venizelou General and Maternity Hospital, Athens, GRC; 2 V Department of Obstetrics/Gynecology, Helena Venizelou General and Maternity Hospital, Athens, GRC; 3 Department of Anesthesiology and Pain Medicine, "Alexandra" General Hospital of Athens, Athens, GRC; 4 Department of Obstetrics and Gynecology, Attikon Hospital, Athens, GRC; 5 Department of Anesthesiology, General and Maternity Hospital of Athens, Athens, GRC

**Keywords:** exploratory laparotomy, acute pelvic pain, leiomyoma, uterine fibroid, parasitic leiomyoma

## Abstract

Parasitic fibroids are a rare type of extrauterine benign tumors that may be spontaneous or iatrogenic in origin and often difficult to diagnose due to their various presentations. We report an unusual case of a parasitic leiomyoma in a 33-year-old nulliparous woman with remote pelvic history who presented to our institution with sudden-onset lower abdominal pain. We performed an exploratory laparotomy, which revealed a 6.3x4.6 cm mass in the space of the adnexa of the right parametrium. Histopathological examination revealed features compatible with a leiomyoma. It is clear that physicians need to assess clinical findings and imaging techniques in order to establish a correct diagnosis of parasitic myomas, even when a history of myomectomy or a laparoscopic morcellation is absent.

## Introduction

Fibroids of the uterus are among the most common benign pelvic tumors that gynecologic oncologists encounter in their clinical practice. They may occur in 20% to 40% of women of reproductive age [1]. These benign neoplasms are composed of smooth muscle cells and fibrous connective tissue. They are classified according to their location in relation to the myometrium; those that develop inside the myometrium are called intramural, if they extend to the serosa they are called subserosal, and, lastly, the submucous protrude in the endometrium cavity [1].

Uterine fibroids are classified into four types by the International Federation of Gynecology and Obstetrics: submucosal, intramural, transmural, and subserosal. Parasitic fibroids are rare extra-uterine benign tumors detected in the abdominal cavity with no relation to the myometrium, grouped as a subtype of subserosal fibroids [2]. They are frequently found in women of reproductive age with a surgical history of laparoscopic myomectomy with power morcellation. Predisposing factors also include age between 40 and 60 years, with a higher prevalence in African American females [1]. Their clinical presentation is quite nonspecific, with most patients being asymptomatic. However, when symptomatic, the most common symptoms include abdominal pain, pressure, or distension [2]. The term “parasitic” has been correlated with a non-disseminating pattern, also attributed to the myomas receiving alternative blood supply from another source besides the uterus, such as the omentum and mesenteric vessels [3].

 A surgical history of laparoscopic morcellation has been associated with parasitic fibroids in recent literature [2]. Spread of the morcellated fragments that were not collected increase the risk of developing parasitic fibroids inside the peritoneal cavity [4].

The most common method of treatment of a parasitic myoma is surgical excision either by laparotomy or under laparoscopic guidance. If during the operation, the surgeons encounter multiple myomas, the differential diagnosis should also include leiomyomatosis peritonealis disseminata (DPL) [2], in which multiple nodules are found in the pelvis, peritoneum, or intestine.

 Herein, we present an unusual case of a parasitic leiomyoma with sudden-onset lower abdominal pain caused by necrotic ischemia in a woman with remote pelvic history.

## Case presentation

A 33-year-old nulliparous (Greek/Mediterranean) woman with regular menstrual cycles was admitted to our emergency department complaining of a 12-hour history of sudden-onset severe right iliac fossa (RIF) pain. Νο episodes of vomiting or any other intercurrent symptoms from the gastrointestinal tract were displayed. Her surgical history included appendicectomy at 17 years of age while her medical history was notable for bleeding disorders due to Willebrand factor deficiency. She was not under contraception medication.

 On examination, the patient’s vital signs were normal (blood pressure of 132/66 mmHg, heart rate of 76 bpm). Vaginal examination did not show any abnormalities. Physical examination revealed lower abdominal rigidity and guarding, with an associated pain on the RIF and right vaginal vault, with negative psoas and Murphy signs. Transvaginal ultrasound scan demonstrated a 6.3 x 4.6 cm mass with decreased blood flow in the location of the adnexa of the right parametrium. Blood tests showed elevated white blood count (18,40 K/μL). Her inflammatory markers were mildly elevated (-reactive protein [CRP]: 42 mg/L), while the metabolic panel was unremarkable (glucose: 80 mg/dL, urea: 14 mg/dL, creatinine: 0.77 mg/dL, SGPT [serum glutamic-pyruvic transaminase]: 16 IU/L, SGOT [serum glutamic-oxaloacetic transaminase]: 24 IU/L). Beta hCG was negative.

**Figure 1:**
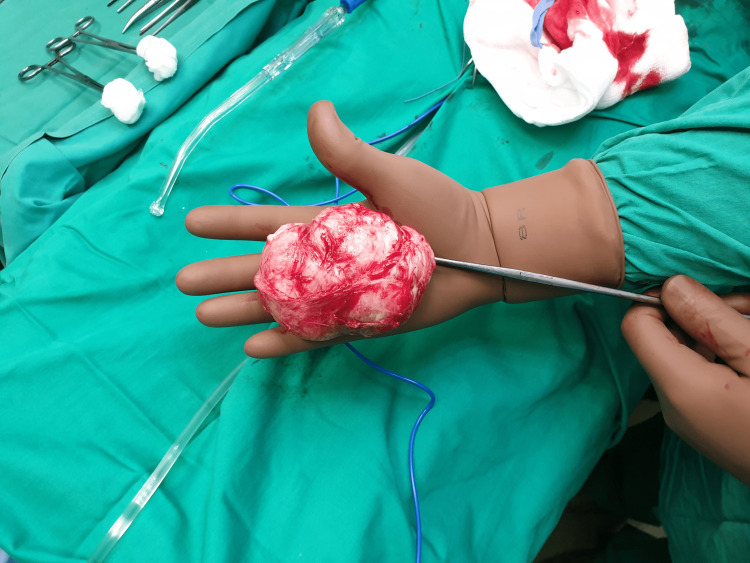
Parasitic fibroid removed from the patient

On the basis of these findings and a high suspicion of adnexal torsion, the patient was taken to the operating theatre and an emergency laparotomy was performed under general anesthesia. Upon entry into the peritoneal cavity, a pedunculated subserosal fibroid was visualized, with what appeared to be a narrow stem attached to the uterus. A small amount of blood-tinged fluid was noted within the pelvic cavity. Multiple adhesions located in the right peritoneum were reported. The adnexa were examined, and no abnormalities were identified. During symphysiolisis, it was revealed that the fibroid’s major blood supply was provided by a large vessel originated from the peritoneum and the fibroid was held in place close to the uterus due to the adhesions. After grasping the myoma, cross-section and ligation of its peritoneal stem was performed. Thus, the fibroid was removed (Figure 1). After complete hemostasis was achieved, an intraperitoneal Penrose drain was installed. The estimated blood loss at the end of the surgery was 500 mL.

Post-operatively, the patient was administered intravenous fluids and broad-spectrum antibiotics. Blood tests performed after the surgery showed a sharp decline of inflammatory markers (CRP: 13 mg/L) and minimal decrease of hemoglobin (9.7 g/dL). The patient’s postoperative course was uneventful, and she discharged home four days later.

At her four-week follow-up visit, the patient was symptom-free. Ultrasound imaging revealed no signs of mass in the cul-de-sac or blood in the peritoneal cavity. Histopathological examination confirmed that the mass was leiomyoma characterized by hyaline necrosis and increased cellularity.

## Discussion

Myomas are a frequent finding in women of reproductive age [5]. Traditionally, parasitic fibroids, mainly caused by extrauterine seeding, were believed to be subserosal benign tumors that while connected to the uterus became attached to adjacent organs, creating alternative vascularization for their blood supply [6]. Another possible explanation could be hormonal, causing metaplasia in the peritoneum, thus explaining the unexpected sites of myomas in the abdomen [7]. The detachment of the subserosal fibroid from the stem connecting it to the uterus serves as the trigger for its creation [8].

The newer hypothesis is that as a byproduct of laparoscopic morcellation of uterine myomas in the past, tissue spread in the abdominal cavity provided the seed for parasitic leiomyomas [2]. Incidence rate of parasitic leiomyomas due to iatrogenic causes has been documented to be between 0.12% and 0.9% [9]. Α safety statement by the FDA issued in 2014 recommended avoidance of laparoscopic morcellation during myomectomies or hysterectomies [8].

Any anatomical structure of the peritoneal cavity may be the site for development of a parasitic myoma. They are usually found in the peritoneum of the pelvic or abdominal wall, omentum, pouch of Douglas, small intestine, or colon [10].

 The diagnosis of a parasitic myoma is challenging since it presents with no special symptoms, and imaging techniques fail to differentiate it from other possible causes [4]. Thankfully, parasitic myomas appear to have low recurrence rate, with only a few documented cases.

Parasitic fibroids are usually treated by surgical removal, either in open surgery or laparoscopically. Since their blood flow can originate from the greater omentum, small intestine, large intestine, or peritoneum [6,11,12], it is important to locate the adjuvant blood supply of the myoma during the operation in order to avoid injury to other organs [2].

In our case, the patient presented with nonspecific symptoms and signs, which impeded a correct diagnosis. According to her medical history, no relevant causes of development of parasitic myomas according to current literature were reported. Since triggering factors causing the development of parasitic fibroids remain unclear, all necessary intraoperative precautions minimizing their formation should be taken.

## Conclusions

Parasitic fibroids are rare commonly presenting with no specific symptoms, making diagnosis difficult. Spontaneous parasitic leiomyomas, along with their complications such as torsion and necrosis, should be placed high in the differential diagnosis in women presenting with sudden-onset lower abdominal pain, especially in those with remote pelvic history of myomectomy or laparoscopic morcellation.
